# Effects of Nanoscale Morphology on Optical Properties of Photoluminescent Polymer Optical Fibers

**DOI:** 10.3390/polym14163262

**Published:** 2022-08-10

**Authors:** Edith Perret, Konrad Jakubowski, Manfred Heuberger, Rudolf Hufenus

**Affiliations:** 1 Laboratory for Advanced Fibers, Empa, Swiss Federal Laboratories for Materials Science and Technology, Lerchenfeldstrasse 5, 9014 St. Gallen, Switzerland; 2 Center for X-ray Analytics, Empa, Swiss Federal Laboratories for Materials Science and Technology, Überlandstrasse 129, 8600 Dübendorf, Switzerland

**Keywords:** polymer optical fiber, light scattering, light conversion, luminescent solar concentrator, semi-crystalline polymer

## Abstract

Bicomponent photoluminescent polymer optical fibers (PL-POFs) have been melt-spun and in-situ drawn to different extents. The results suggest that scattering in the sheath can effectively increase the photoluminescent dye excitation probability in the fiber core. The core/sheath PL-POFs are made of a semi-crystalline fluoropolymer sheath of low refractive index (RI) and an amorphous cycloolefin polymeric core of high RI, which is doped with a luminescent dye. The axial light emission, as well as the guiding attenuation coefficients of the core/sheath PL-POFs, have been measured using a side-illumination set-up. The incident blue laser is down-converted to red light, which is re-emitted and partially guided by the core. The axial light emission is measured at the fiber tip as a function of the distance from the illumination position to the integrating sphere. It is demonstrated that the presence of a semi-crystalline sheath significantly enhances the axial light emission and that it also lowers the attenuation coefficient, compared to the emission and guiding properties of PL core-only fibers. Additionally, the attenuation coefficient has been found to be lower in more strongly drawn PL-POFs. Wide-angle X-ray diffraction and small-angle X-ray scattering experiments reveal structural differences in differently drawn PL-POFs that can be linked to the observed differences in the optical properties.

## 1. Introduction

**Figure 1:**
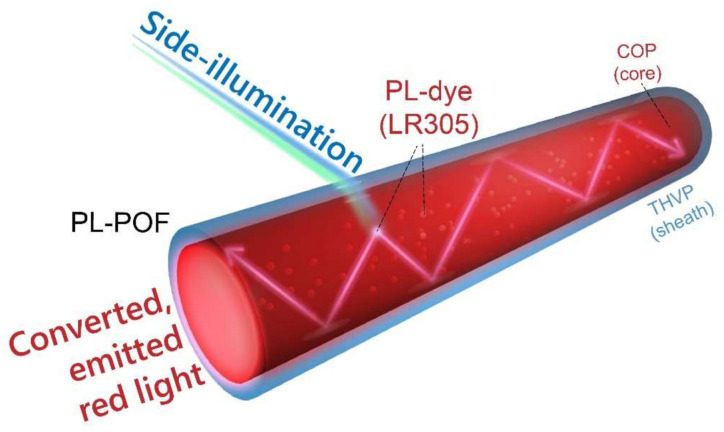
Down-conversion of incoming light from side-illumination of a PL-POF into axially emitted red light. The PL dye (LR305) in the core of the PL-POF is responsible for the light conversion from blue to red. Reprinted/adapted image with permission from [15]. 2020, Elsevier.

Luminescent waveguides [1,2,3] have been successfully utilized as luminescent solar concentrators (LSCs) for photovoltaic cell enhancement [4,5,6], to amplify signal detection of free-space optical communication systems [7,8,9], or to design color-tunable decorative elements [10]. Luminescent waveguides, in the form of polymer optical fibers (POFs) [2,11], can be produced by melt-spinning [12,13,14], which is the most economical fiber spinning process. Photoluminescent (PL)-POFs contain a high refractive index (RI) polymer core, which is doped with a PL dye that down-converts (change of wavelength) and emits light. Typically, such a core is enclosed within a low-RI polymer sheath, since the sheath provides enhanced mechanical as well as optical stability. Note, that for bicomponent fibers, the material that surrounds the core is typically called ‘sheath’ and for POFs, it is sometimes also called ‘cladding’. In the remainder of this manuscript, the surrounding material is called ‘sheath’. In contrast to standard optical fibers, PL-POFs are able to collect light to a great extent from side-illumination and convert and guide the light to the tip of the fiber. An example of such a device is shown in Figure 1. A photoluminescent dye, Lumogen Red 305 (LR305), which is doped to the core of a PL-POF, absorbs blue light from side-illumination and emits down-shifted red radiation, which is guided axially towards the tip of the PL-POF by the total internal reflection at the core-sheath interface [15] (Figure 1). In the past, we have studied the luminescent properties of such PL-POFs for different core host materials, as well as optical conversion efficiencies for fiber bundles as a function of illumination angle [15].

The present motivation in the field is to optimize the optical properties of photoluminescent waveguides. Especially the intensity of luminescence measured at the fibers’ tip (axial emission) has been intensively studied in the past, since it defines the effectiveness of dope-dyed waveguides, e.g., in solar concentrator applications. A strategy to improve the axial emission is to maximize the interaction of incident light with the PL containing medium, by increasing the geometric optical path within the waveguide. This can be achieved by making use of light scattering, which is introduced in the form of scattering centers either outside or inside the waveguides. For example, to improve the emission intensity of planar LSCs, the excitation light has been passed through an additional scattering or diffusing frontal layer, thus increasing its interaction path with the PL containing medium [16,17,18,19]. Furthermore, adding scattering microspheres directly into the luminescent waveguide can also increase the performance of such LSCs [20]. The latter method is, however, accompanied by higher propagation losses, since microspheres also impede the guidance of the light. In polymeric waveguides [21], such as heat-processed polymer plates, the light scattering arises from crystalline structures (large spherulites) within the amorphous phase [22,23,24]. Different densities, and thus different RIs, of crystalline and amorphous regions constituting a semi-crystalline polymer [25,26] are responsible for the scattering of light. In the case of POFs, the thinness of the sheath material hinders the formation of large spherulites but smaller crystals, called lamellae, can be formed [27].

In this work, bicomponent melt-spinning [13] was utilized to prepare PL-POFs (as shown in Figure 1), where both the core and the sheath are processed simultaneously into a continuous filament [12,28,29,30,31,32]. Two PL-POFs of different draw ratios (i.e., the ratio between the winder and first take-up godet speed in m/min) have been prepared. The aim of this work was to study the impact of the semi-crystalline morphology of the sheath material on the axially emitted photoluminescence intensity (axial emission). PL-POFs serve as a benchmark and are cross-referenced with core-only fibers in order to isolate the effect of the scattering from the semi-crystalline sheath material onto the axial light emission. For this purpose, axial light emission was measured by side-illuminating both core/sheath PL-POFs as well as core-only PL-POFs with a blue laser. Using this side-illumination setup, it was possible to measure the axial emission from the fibers’ tip as a function of the distance from the illumination position and thus also determine axial guiding attenuation coefficients. In addition to axial light emission measurements, the nano-structural properties have been determined by measuring wide-angle X-ray diffraction (WAXD) and small-angle X-ray scattering (SAXS) [33,34,35,36,37,38] patterns of core/sheath and core-only fibers. Differences in the structural properties of PL-POFs due to different draw ratios are discussed and correlated to differences in axial light emissions.

## 2. Materials and Methods

### 2.1. Materials

Several thermoplastic polymers are suitable and commercially available [39,40] for a PL-POF core. We have chosen the cycloolefin (COP) polymer, which was previously identified as the best host material for a specific photoluminescent dye, Lumogen Red 305 (LR305), as the formation of dye aggregates is less pronounced in this material [15]. COP granulates (Zeonor 1020R, refractive index 1.53) were purchased from Zeon Europe GmbH, Düsseldorf, Germany. The photoluminescent dye, LR305 (BASF, Basel, Switzerland), was added to the COP core at a concentration of 0.01 wt.%. The terpolymer of tetrafluoroethylene, hexafluoropropylene and vinylidene fluoride polymer (THVP), has become a common choice for the sheath material of melt-processed POFs [12,15,29,31]. THVP is characterized by good transparency, low RI, good melt-spinnability, and semi-crystallinity [41]. THVP granulates (THVP 2030GZ, RI = 1.35) were purchased from 3M GmbH, Rüschlikon, Switzerland. Acetone and methoxyperfluorobutane were purchased from Sigma Aldrich, Buchs, Switzerland, in order to remove the sheath for subsequent studies of the optical and structural properties of the core material. All materials were used as provided without purification.

### 2.2. Bicomponent Melt-Spinning of PL-POFs

**Figure 2:**
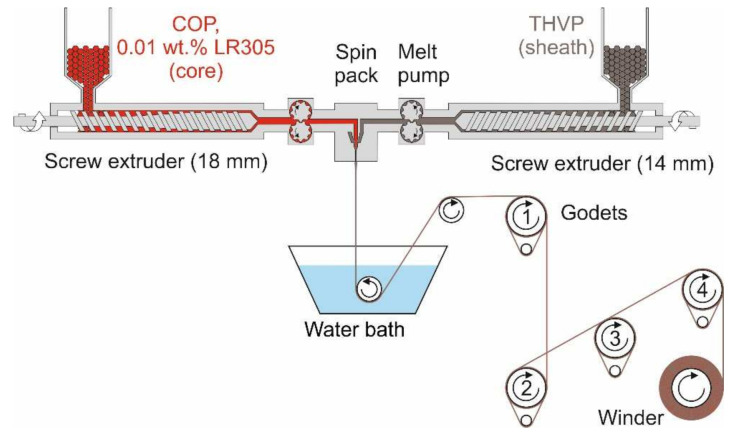
Schematic of pilot melt-spinning plant used to prepare PL-POFs.

A bicomponent melt-spinning procedure [14,15] was used to prepare core/sheath PL-POFs with a COP core and a low-RI, semi-crystalline THVP sheath. The melt-spinning setup is illustrated in Figure 2.

Previously dried COP (core material) was tumble-mixed with 0.01 wt% LR305 and fed into an 18 mm diameter extruder. The extruder encompassed three heating zones, which were set to 180, 265 and 270 °C. The melt was then pumped into the spin pack at 10.5 cm^3^/min. The THVP (sheath material) was fed into a 14 mm extruder, which had three heating zones set to 100, 165 and 280 °C. The melt was pumped into the spin pack at a rate of 6 cm^3^/min. The fibers were melt-spun into a water bath followed by a drawing over four godets in series (Table 1). Two different draw ratios (ratio between the winder and take-up godet 1 speeds in m/min) have been applied, namely DR = 1.1 and DR = 1.6. The maximum achievable draw ratio for the POFs was limited by the amorphous core material, since stretching beyond 1.6 caused draw-induced microcracks.

### 2.3. Sheath Removal from POFs

In order to study the axially emitted photoluminescence intensity as well as the structure of the COP core alone (sheath removed), it was necessary to expose the COP core by dissolving the THVP sheath [42]. For this purpose, the fibers were immersed in a solution of acetone and methoxyperfluorobutane in equal proportions for 90 min at room temperature. The solution was constantly stirred during this procedure. The fibers were then removed from the solution and rinsed to remove any residues of THVP, and finally dried in air. Spectrophotometric control verified that the waste solution did not show any dye coloration, indicating that the procedure was selective on the sheath material and did not dissolve the dye-doped COP core. Fibers with removed sheath are labeled as ‘core-only’ in the remainder of the manuscript.

### 2.4. Axial Light Emission Measurements

**Figure 3:**
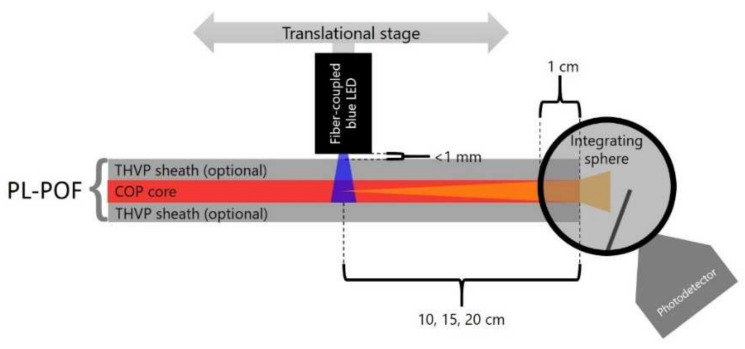
Schematic representation of the setup used to measure the photoluminescence intensity at a defined distance, *x*, from the side-illumination point.

To assess the efficiency of collecting the light from side-illumination and guiding it to the fiber tip, we have measured the axially emitted photoluminescence intensity with an integrating sphere, which was attached to a photomultiplier tube (Figure 3). Core/sheath or core-only fibers were fixed in a straight line, with one end being inserted inside the sphere, and fixed at its entrance with a small stripe of double-sided tape. To minimize light absorption in contact with the adhesive tape, especially for exposed fiber cores, a very small stripe (<1 mm) was used. A blue light-emitting diode (LED) with an emission peak at 455 nm (M455F3, Thorlabs, Germany) illuminated the fiber orthogonally to its axis via a glass optical fiber (M28L02, Thorlabs, Germany). Note that the fiber diameter is small compared to the absorption length within the polymer core at the excitation source wavelength. Thus the illuminated portion of the fiber can be approximated as a homogenous light source (plane-wave source) [43,44]. The LED was re-positioned along the fiber axis and the intensity of emitted photoluminescence was measured at selected distances (*x* = 10, 15 and 20 cm) between the LED and the integrating sphere (Figure 3).

To compare measured intensities of differently drawn core/sheath PL-POFs with different diameters, a normalized specific intensity (NSI) was defined. The normalized specific intensity divides the measured intensity by the cross-sectional area of the fiber core.

(1) $$NSI\left(x\right)=\frac{I_{\mathrm{measured}}\left(x\right)}{A_{\mathrm{core}}\;}$$

*I*_measured_(*x*) is the measured light intensity for a specific distance, *x*, of the LED from the integrating sphere, and *A*_core_ is the cross-sectional area of the luminescent core. Note, that a correction accounting for trapping efficiencies [45] of core/sheath and core-only fibers, which are solely based on refractive indices of core and sheath materials, is intentionally avoided here. This way the observed differences in NSI values of core/sheath PL-POFs compared to core-only fibers can isolate the effect of the sheath material on the axial light emission.

Translation of the illumination point to at least two different distances from the emitting fibers’ tip allows calculation of the attenuation coefficient in [dB/m], in analogy to the so-called cut-back technique [41]:

(2) $$\alpha =-\frac{10}{\Delta d}\log\left(\frac{I_{20}}{I_{10}}\right)$$

Here, *I*_20_ and *I*_10_ are the intensities measured at positions 20 cm and 10 cm away from the fiber tip, respectively, and Δd=0.2 m−0.1 m=0.1 m is the distance between these positions.

### 2.5. X-ray Measurements

Wide-angle X-ray diffraction (WAXD) and small-angle X-ray scattering (SAXS) patterns were measured from core/sheath and core-only fibers (without THVP sheath) in order to determine meso- and nano-scale structural details arising from the core and sheath, respectively. WAXD and SAXS patterns were recorded on a Bruker Nanostar U diffractometer (Bruker AXS, Karlsruhe, Germany) with a beam-defining pinhole of 300 μm, with Cu Kα radiation (λ = 1.5419 Å) and a VÅNTEC-2000 MikroGap area detector. WAXD and SAXS measurements were performed in two separate experiments with distances of 9.4 cm and 110.8 cm between the fiber and the active detector area, respectively. Single fibers were mounted on holders for all measurements. WAXD patterns have been recorded for 30 min and SAXS patterns for 77 min.

## 3. Results and Discussion

### 3.1. Dimensions of Core/Sheath PL-POFs

**Figure 4:**
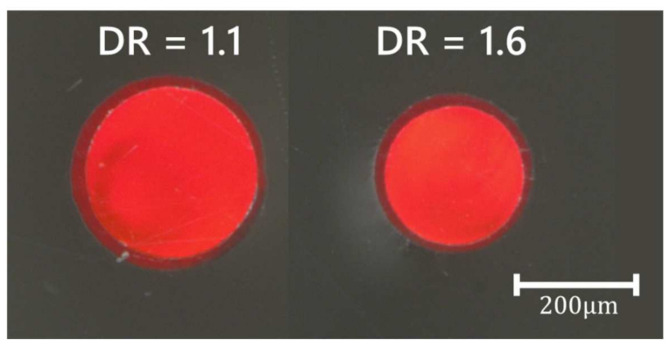
Microscopic cross-sections of core/sheath PL-POFs (DR = 1.1 and DR = 1.6) with doped COP core and pure THVP sheath.

Microscope images (VHX-1000D, Keyence, Osaka, Japan) of the core/sheath PL-POFs with DR = 1.1 and DR = 1.6 are shown in Figure 4. The determined fiber dimensions (core radii, core areas, sheath thickness, outer PL-POF radii and PL-POF areas) are summarized in Table 2.

The ratio of the core area to the total fiber cross-section area is around 76% (diameters scale linearly under drawing). The average values and standard deviations of the fiber dimensions given in Table 2 have been determined from five measurements.

### 3.2. Axial Emission Properties

In order to isolate the impact of the semi-crystalline sheath material (THVP) on the axially emitted photoluminescence intensity, the light emission of both the core/sheath PL-POFs as well as of the core-only fibers have been compared. Photoluminescence intensities, *I*_measured_ (x), normalized specific intensities, *NSI*(*x*) (Equation (1)), and attenuation coefficients, α (Equation (2)), have been determined with the optical emission method described in Section 2.4 (Figure 3). The measurements have been repeated five times and average values, as well as standard deviations, are summarized in**Table 3**. Note, that the concentration of the photoluminescent dye, LR305, in the COP core is for all fibers 0.01 wt.%. Thus, the magnitude of self-absorption (overlap of emission with absorption band) is expected to be identical in all fibers.

#### Impact of the Presence of a Semi-Crystalline Sheath Material onto Axial Emission

The following observations are made when comparing the optical properties of core/sheath PL-POFs with core-only fibers (Table 3):

- NSI values of core/sheath PL-POFs are significantly higher than NSI values of core-only PL-POFs. These values depend on the light-incoupling and light-guiding ability of the fiber.
- The attenuation coefficients of core/sheath PL-POFs are smaller than the ones of core-only PL-POFs. The smaller the attenuation coefficient, the less light is lost over a fixed distance along the fiber. Thus, the removal of the sheath material for core-only fibers induces additional attenuation.

The calculated *NSI*(*x*) from the measured intensities has been fitted to the following empirical function:

(3) $$NSI_{fit}\left(x\right)=NSI\left(0\right)\times (10^{-\frac{\alpha }{10}\cdot x})$$

**Figure 5:**
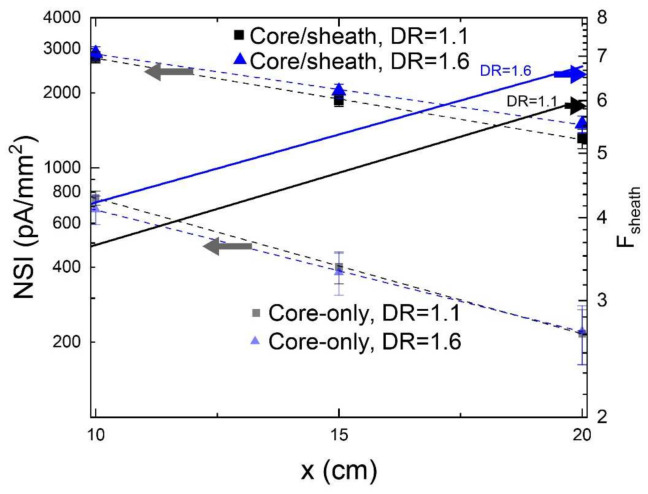
Logarithmic plot of NSI-values and calculated sheath-related improvement parameters, F_sheath_, for PL-POFs of DR = 1.1 and DR = 1.6. The fitting curves that are based on Equation (3) are shown as dashed curves.

*NSI*(0) is the normalizing specific intensity, which is a fitting parameter. The fitting function also includes the attenuation constant α (Equation (2)), and as boundary condition, the three calculated *NSI*(*x*) values from Equation (1) for *x* = 10, 15 and 20 cm. In Figure 5, the calculated *NSI*-values are plotted logarithmically together with the fitting curves (dashed lines) from Equation (3). The improvement parameter due to the sheath, *F*_sheath_, is calculated as follows:

(4) $$F_{sheath}=NSI_{fit}^{core/sheath}\left(x\right)/NSI_{fit}^{core-only}\left(x\right)$$

This parameter, *F*_sheath_, reflects the improvement of the normalized axial emission at the fibers’ tip due to the presence of the sheath material; it is also a function of the distance of the illumination point to the detector. Note, that the *F*_sheath_ parameters are plotted logarithmically in Figure 5 on the right axis. For both types of PL-POFs, the parameter increases with increasing distance, *x*, and ranges from 3.7 to 6.1 (*x* = 10 to 20 cm) and from 4.2 to 6.8 for DR = 1.1 and 1.6, respectively.

To summarize, the sheath improvement parameters reveal that a semi-crystalline sheath material can provide a significant improvement in the normalized specific emission intensity. Note, that a similar effect of the sheath onto the axial emission has been observed for other types of PL-POFs, e.g., for a thermally drawn PL-POF, where the addition of a 20 µm thick, solution-processed acrylate sheath has been shown to improve the axial emission by a factor of 2.6, compared to the core-only fiber [46].

**Figure 6:**
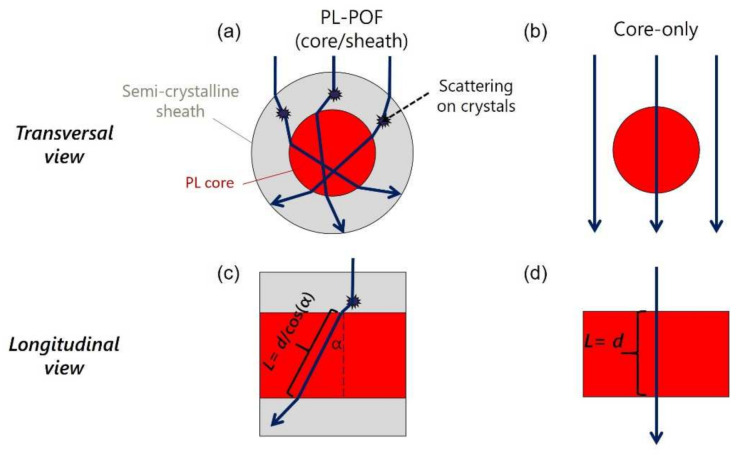
Schematic explanation of the effect of scattering in a semi-crystalline sheath onto axial light emission. The transversal transmission of light through a core/sheath PL-POF is shown in (**a**) and through a core-only fiber in (**b**). Corresponding longitudinal views are shown in (**c**,**d**). In summary, scattering in the sheath leads to prolonged optical pathways across the photoluminescent core, thus increasing the probability for light down-conversion with axial re-emission.

Higher NSI values (axial emission) of core/sheath PL-POFs, compared to core-only fibers, arise mainly due to differences in the in-coupling of the light. For core/sheath PL-POFs, the NSI values depend on the refraction events at the air/sheath interface and scattering events that take place in the sheath material due to the presence of crystals. Figure 6 schematically explains the improvement of the axial emission intensity due to the semi-crystalline sheath material. Refraction at the air/sheath interface and scattering events in the sheath material lead to prolonged optical pathways across the photoluminescent core, thus increasing the probability for light down-conversion with axial re-emission. Small crystals and crystalline domains in the sheath material act as light-scatters because of the discontinuities in the refractive index at the amorphous/crystalline phase boundaries. Various types of optical scattering phenomena can occur depending on the size of the scattering centers. For example, Rayleigh scattering occurs due to the presence of crystals that are much smaller than the incident wavelength (crystal diameter *D* < 1/10 λ, *D* < 45.5 nm) [47]. Mie-scattering, however, arises from micro-domains in the polymer sheath [25,47,48]. Mie-scattering becomes more intense in the forward direction and its scattering intensity increases with the size of the crystalline domains. In the case of Rayleigh scattering, however, the backward and forward scattering are similar in intensity. Along these lines, the nanostructure of the PL-POFs has been investigated with X-rays as discussed in Section 3.3 below.

Note, that in Figure 6 the absorption and re-emission events, due to the presence of the PL dye in the core, are not shown, since the focus lies on comparing the pathway of the optical light, which reflects the probability of the light interacting with the dye. Due to the complexity arising from a large number of possible light rays (refraction, scattering events), the schematic in Figure 6 is only a simplified representation of possible pathways of light rays. Two perspectives are considered:

- A 2D-view of the transversal cross-section of a core/sheath PL-POF (Figure 6a) and a core-only fiber (Figure 6b). The incoming light is refracted at the air/sheath interface and crystals in the sheath material of a PL-POF deflect the incoming light in different directions. Thus in the case of PL-POFs, a large part of the incoming light interacts with the core. Note, that light that is transmitted transversely through the fiber can also be returned to the core via scattering events. In contrast, for core-only fibers (Figure 6b) some light rays pass the fiber without even interacting with the core.
- A 2D-view of the longitudinal cross-section of a core/sheath PL-POF (Figure 6c) and a core-only fiber (Figure 6d). Forward-scattering crystals in the sheath material of a PL-POF deflect the light path, resulting in longer traveling distances, *L*, of the light inside the core, thus a higher probability of dye excitation. In contrast, the undeflected wave in a core-only fiber (Figure 6d), has an interaction path that is maximally equal to the fibers’ diameter *d*.

It is concluded that in the case of PL-POFs, the refraction events at the air/sheath interface and scattering events in crystals in the sheath material led to increased axial emission values due to the fact that more light is guided into the core, enhancing the PL dye excitation.

The optical measurements also show that the sheath material in PL-POFs is essential to minimize attenuation losses. The following factors may be the cause of lower attenuation coefficients of the core/sheath PL-POFs compared to core-only PL-POFs:

(1) During sheath removal, the solvent may cause microcracks in the surface of the amorphous core, which in turn increases the attenuation [49].
(2) In core/sheath PL-POFs, a robust optical interface between the two materials exists, making the waveguide less sensitive to the negative impact of external factors, such as dust particles that attach to the surface.

In PL-POFs, excitation light rays that leave the core (‘lost’ light) and do not fulfill the total internal reflection condition, are refracted at the core/sheath interface and enter the sheath material. For a semi-crystalline polymer material, these short-wavelength rays can be back-scattered into the core through, e.g., Rayleigh scattering due to crystals of a few tens of nanometers in size [25,48]. This back-scattering mechanism is particularly effective for the short-wavelength excitation light since the intensity of the scattered Rayleigh radiation is proportional to 1/λ^4^. Therefore, blue light is even more strongly back-scattered into the core than red light. These rays of blue light can then excite the dye and be down-converted to red light and re-emitted into guiding modes of the core.

Weiss [45] has put forward an equation to predict the trapping efficiencies of fluorescent core/sheath POFs. The trapping efficiency is defined by the ratio between the trapped (or guided) optical energy and the total energy emitted from fluorescence in the fiber core and it can be derived from the refractive indices, *RI*, of the core and sheath materials [45]:

(5) $$\eta_{\mathrm{tr}}=1-{\left(\frac{RI_{\mathrm{sheath}}}{RI_{\mathrm{core}}}\right)}^{2}$$

For PL-POFs, the refractive indices are *RI*_sheath_ = 1.35 for the THVP sheath and *RI*_core_ = 1.53 for the COP core material, respectively. In the case of core-only fibers, however, the surrounding *RI* = 1.0 arises from the air. The calculated trapping efficiencies of the core/sheath POF is *η*_tr,POF_~0.22 and the calculated trapping efficiency of the core-only fiber is *η*_tr_core-only_~0.57. These values would suggest that thanks to a higher RI difference, core-only fibers are able to trap and guide more light to the end of the fiber tip than core/sheath fibers. This is in contradiction to our findings from optical measurements of the axial light emission, where we have observed the opposite trend; the PL-POFs show larger axial emission values than core-only fibers. The experimental results strongly indicate that it is necessary to take into account additional parameters, such as the ability of crystals in the sheath material to scatter and couple light into the core. As is shown by the experimentally observed sheath-induced improvement parameter, the presence of crystals in the sheath can significantly enhance the in-coupling of the light, as well as enhance the back-scattering of blue light into the core and thus effectively increase the optical trapping efficiency.

### 3.3. Structural Properties

**Figure 7:**
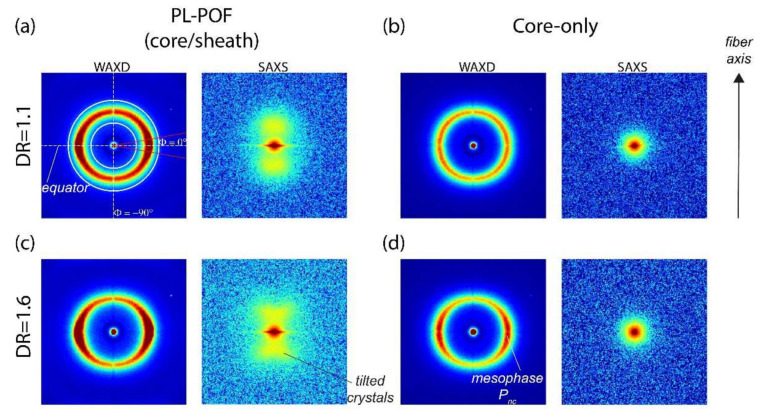
WAXD and SAXS patterns for (**a**) PL-POF with DR = 1.1 and corresponding (**b**) core-only fiber. WAXD and SAXS patterns for (**c**) PL-POF with DR = 1.6 and corresponding (**d**) core-only fiber. The fiber axis is vertical.

**Figure 8:**
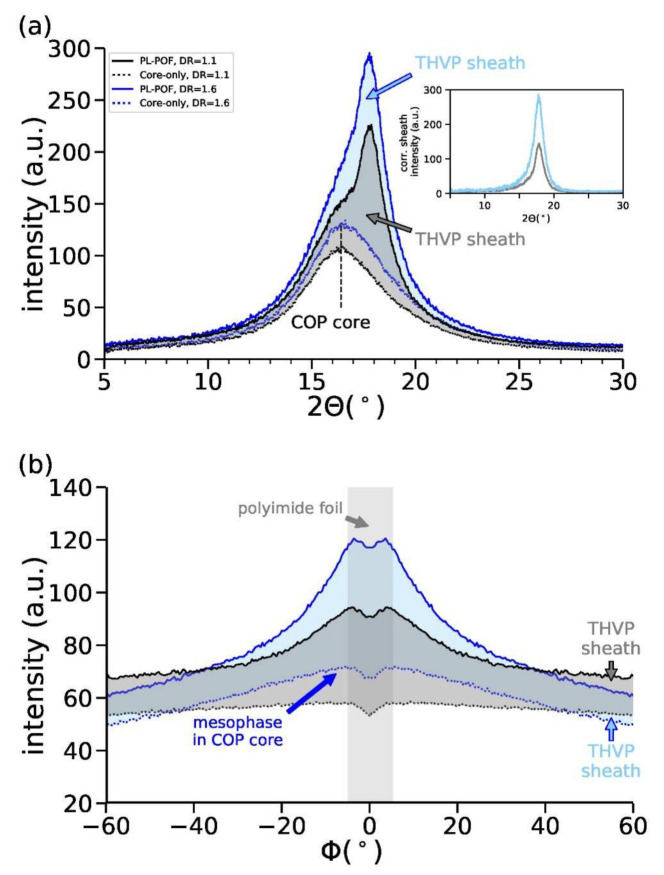
(**a**) Equatorial WAXD profiles and (**b**) azimuthal profiles of PL-POFs and corresponding core-only fibers. The inset in (**a**) shows the corrected equatorial intensity of the sheath material, which is obtained by subtracting the intensity of the core-only fiber from the PL-POF. The correction is explained in the main text. The intensity in the central region of (**b**), around ϕ = 0°, is slightly decreasing due to a polyimide foil that holds the beamstop.

To further characterize the crystalline morphology of the sheath that likely affects light scattering, we have performed WAXD and SAXS measurements on PL-POFs and core-only fibers. The measured (X-ray) scattering patterns are shown in Figure 7 for DR = 1.1 and DR = 1.6. The fiber draw ratio is expected to directly affect the crystalline morphology. A comparison of the WAXD and SAXS patterns of PL-POFs with the corresponding core-only fibers reveals that both PL-POFs consist of an amorphous core (COP) and a semi-crystalline sheath (THVP). This conclusion is drawn from the fact that SAXS patterns of core-only fibers (Figure 7b,d) do not show any scattering features arising from lamellar crystals, whereas SAXS patterns from PL-POFs show straight or tilted reflections (Figure 7a,c). The complete absence of the lamellar reflections in the SAXS patterns of the core-only fibers proves that the sheath has been completely removed. Additionally, the WAXD patterns of core-only fibers show a typical halo (Figure 7b), which reflects the amorphous nature of the COP core material. Interestingly, the WAXD pattern of the core-only fiber DR = 1.6 in Figure 7d shows an additional arc-shaped equatorial reflection, which signifies that part of the COP polymer chains became partially oriented along the fiber axis due to the higher draw ratio [29]. Such arc-shaped equatorial reflections have already been observed in different types of amorphous and also semi-crystalline polymer fibers in the past and have been identified to arise from a highly-oriented non-crystalline mesophase, P_nc_ [34,35,36,50]. To better visualize the differences between WAXD patterns from PL-POFs and core-only fibers, as well as between differently drawn fibers, we have extracted equatorial and azimuthal profiles. The equatorial profiles have been extracted by an azimuthal integration over ± 10° (see equatorial sector between red lines in Figure 7a). The azimuthal profiles are obtained by integrating between 2*θ* = 11–22° (see white rings in Figure 7a). All profiles are shown in Figure 8 and have been normalized by the core areas to account for the differences in scattering volume. Thus, the intensities arising from the THVP sheaths correspond to the difference in intensities between the profiles from PL-POFs and the corresponding profiles from core-only fibers. These differential intensities are shaded in grey and blue in Figure 8, respectively. PL-POFs exhibit a pronounced peak at around 2*θ*~17.7° on the equator (Figure 8a), which arises from crystals in the THVP sheath. This equatorial peak is more pronounced for fibers with DR = 1.6 due to the overlap with the underlying equatorial mesophase signal (Figure 8b, dotted blue curve) [13] and an increased crystallinity. The higher crystallinity of the sheath material in the PL-POF with DR = 1.6, compared to the PL-POF with DR = 1.1, is reflected by the difference in equatorial intensities of the PL-POFs and core-only fibers (inset of Figure 8a). Note, that the displayed sheath intensity of the PL-POF DR = 1.6 has been divided by its sheath area and multiplied by the sheath area of PL-POF DR = 1.1. Therefore, the difference in the intensities directly reflects the difference in crystallinity of the sheath.

**Figure 9:**
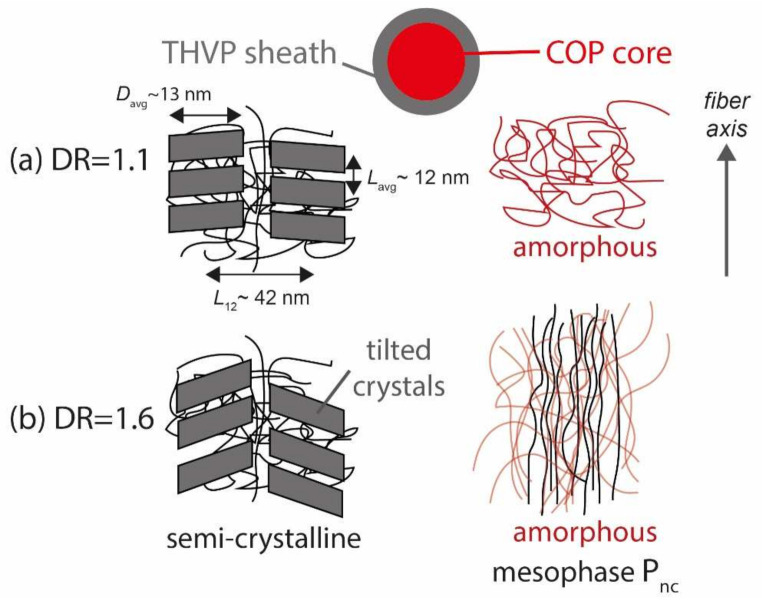
Proposed structure of THVP sheath and COP core for (**a**) DR = 1.1 and for (**b**) DR = 1.6.

An illustration of plausible structures of the THVP sheath and COP core material for the PL-POF fibers, DR = 1.1 and DR = 1.6, from the WAXD and SAXS patterns is shown in Figure 9. The THVP sheath of the PL-POF DR = 1.1 shows a straight ‘two’-point reflection SAXS pattern (Figure 7a), which arises from stacked parallel crystals that are arranged along the fiber axis (Figure 9a, left) [41]. Note, that in reality there are four reflections present in the SAXS pattern; two in the upper half of the SAXS image, which overlap along the transversal direction, and two overlapping reflections in the lower half of the SAXS image. The average long spacing (*L*_avg_~12 nm, Figure 9a, left) between the crystals along the fiber axis is estimated from the location of the reflections along the meridian (parallel to the fiber axis) [51]. The lateral position of the reflections reflects the average lateral spacing between crystals, *L*_12_~42 nm (Figure 9a, left). The width of the crystals, perpendicular to the fiber axis, *D*_avg_ (Figure 9a, left), varies and is estimated from the widths of the reflections to be on average around 13 nm [52,53]. The crystal average size suggests that Rayleigh scattering of visible light is indeed occurring in the sheath material. The WAXD pattern (Figure 7b) shows a halo, thus the COP core is amorphous (Figure 9a, right). For PL-POFs DR = 1.6, the SAXS reflections (Figure 7c), arising from crystals in the THVP sheath, are tilted away from the fiber axis and form an x-shaped four-point pattern. Such x-shaped patterns indicate that large parallel crystals become tilted away from the fiber axis and different long-spacings are introduced between these crystals [33,54,55] (Figure 9b, left). The COP core shows both an amorphous phase and mesophase (Figure 9b, right) in the WAXD pattern (Figure 7d).

### 3.4. Structure-Property Relationship Comparing Differently Drawn PL-POFs

In order to investigate whether normalized scattering intensities or attenuation coefficients for PL-POFs of different draw ratios (Table 3, Figure 5) are significantly different from one another, we have performed statistical Welch tests [56]. Average values are considered to be significantly different when the calculated *p*-value is smaller than 0.05 for a two-tailed test. It is found that the PL-POF with DR = 1.6 has

(i) a significantly lower attenuation coefficient (α = 28.4 ± 4) than the PL-POF with DR = 1.1 (α = 32.6 ± 4), *p* = 0.012.
(ii) a significantly higher NSI value (NSI = 1499 ± 117) than the PL-POF with DR = 1.1 (1312 ± 117) for *x* = 20 cm, *p* = 0.036.

Using WAXD and SAXS one can thus show that the following structural properties are different comparing PL-POFs DR = 1.6 with DR = 1.1; (i) the core of the PL-POF DR = 1.6 contains a mesophase, (ii) the crystallinity of the sheath is higher for DR = 1.6 than for DR = 1.1, and (iii) the crystals in the THVP sheath are tilted. All these structural differences may be responsible for the differences in the axial emission properties of PL-POF DR = 1.6.

Note, that the axial light emission and attenuation coefficients of differently drawn core-only fibers are not significantly different from one another (*p* > 0.05), which suggests that the presence of a mesophase in core-only fibers with DR = 1.6 is not significantly affecting the optical emission properties of the fibers. Therefore, the structural differences of the sheath material in differently drawn PL-POFs have to be mainly responsible for the differences in the optical emission properties. The slightly increased crystallinity in the sheath material of the PL-POF with DR = 1.6 may cause more (blue) light to be scattered back into the core through Rayleigh scattering, compared to sheath materials with lower crystallinity. Note, that the absolute crystallinity is difficult to be reliably determined using X-ray scattering, but for melt-spun semi-crystalline fibers, crystallinity values are typically below 50%.

## 4. Conclusions

PL-POFs are promising materials for light concentrators, luminescent assemblies, or decorative light-emitting textiles [10]. As such, their characterization as leaky, scattering waveguides is of eminent importance. In this article, core/sheath PL-POFs and corresponding core-only fibers have been side-illuminated with a blue laser and the axial light emission was measured. It was demonstrated that the addition of a semi-crystalline sheath can significantly improve the axial light emissions and lower attenuation coefficients of core/sheath PL-POFs compared to PL core-only fibers. A sheath-related improvement parameter was introduced that increased as a function of the distance of the side-illumination point to the integrating sphere. Refraction events at the air/sheath interface and scattering events at crystals in the sheath material have been identified to significantly enhance the in-coupling of blue light from side-illumination into the core, which in turn enhances the PL dye excitation probability leading to higher axial light emission values of PL-POFs. This finding is fundamentally remarkable since in conventional POFs, the scattering of light by the nanostructure of the sheath has no significant effect on the axial light emission due to the lack of light-conversion in PL dyes.

WAXD and SAXS experiments have confirmed that the THVP sheath is indeed semi-crystalline. The crystals in the sheath material have been found to be responsible for the Rayleigh scattering of blue light since the crystals have on average a width (perpendicular to fiber axis) of about 13 nm. Additionally, ‘lost’ blue light (light that leaves the core at angles different from the critical total reflection angle) can be scattered back into the core by the crystals in the sheath through Rayleigh or Mie scattering, enhancing the PL dye excitation probability and thus lowering the attenuation coefficients.

In this work, we have given an overview of possible reasons for the increased axial light emission under the side-illumination of core/sheath PL-POFs. To fully understand the exact mechanisms of how the semi-crystalline sheath (crystal sizes, orientation, crystallinity, sheath thickness, etc.) affects the light-incoupling and back-guiding, further extensive trials and analytics of different types of PL-POFs would have to be performed. For future studies, it would also be of interest to investigate the cross-sectional microstructure of the THVP material with, e.g., high-resolution Raman mapping [57], since crystalline domain sizes that are similar to the wavelength are expected to lead to Mie scattering.

## Figures and Tables

**Table 1:** Production parameters of core/sheath PL-POFs.

Fiber Number Used at Empa	Label	Godet 1 Speed/ Temp. (m/min, °C)	Godet 2 Speed/ Temp. (m/min, °C)	Godet 3 Speed/ Temp. (m/min, °C)	Godet 4 Speed/ Temp. (m/min, °C)	Winder Speed (m/min)	Draw Ratio
1815	Core/sheath 1.1	140/54	140/60	147/30	150/30	148	1.1
1817	Core/sheath 1.6	140/54	140/60	223/30	225/30	223	1.6

**Table 2:** Dimensions of PL-POFs.

Label	Core Radius (µm)	$A_{core}$ (mm^2^)	Sheath Thickness (µm)	Outer PL-POF Radius (µm)	*A*_PL-POF_ (mm^2^)
Core/sheath, DR = 1.1	144 ± 3	0.065	21 ± 6	165 ± 3	0.086
Core/sheath, DR = 1.6	118 ± 4	0.044	17 ± 7	135 ± 3	0.057

**Table 3:** Overview of measured and calculated optical parameters for core/sheath PL-POFs and core-only fibers with two different DRs. The error in the attenuation has been calculated using error propagation.

Fiber	x (cm)	*I*_measured_ (*x*) (pA)	α (dB/m)	NSI(*x*) (pA/mm^2^)
Core/sheath, DR = 1.1	10	181 ± 10	32.6 ± 4	2780 ± 146
	15	121 ± 6		1861 ± 98
	20	85 ± 8		1312 ± 117
Core-only, DR = 1.1	10	49 ± 3	54.4 ± 11	757 ± 48
	15	26 ± 4		401 ± 58
	20	14 ± 3		216 ± 53
Core/sheath, DR = 1.6	10	126 ± 8	28.4 ± 4	2881 ± 178
	15	89 ± 6		2034 ± 134
	20	66 ± 5		1499 ± 117
Core-only, DR = 1.6	10	30 ± 4	48.9 ± 13	682 ± 89
	15	17 ± 3		382 ± 73
	20	10 ± 3		221 ± 59

## Data Availability

The data presented in this study are openly available in the Mendeley repository at DOI:10.17632/84ck2b45h9.1.
